# Identification and Validation of Immune-Related Methylation Clusters for Predicting Immune Activity and Prognosis in Breast Cancer

**DOI:** 10.3389/fimmu.2021.704557

**Published:** 2021-06-30

**Authors:** Anli Yang, Ying Zhou, Yanan Kong, Xiaoli Wei, Feng Ye, Lijuan Zhang, Xian Zhong, Mingyue Li, Shilin Lu, Xin An, Weikai Xiao

**Affiliations:** ^1^ Department of Breast Oncology, State Key Laboratory of Oncology in South China, Collaborative Innovation Center for Cancer Medicine, Sun Yat-sen University Cancer Center, Guangzhou, China; ^2^ Department of Infectious Diseases and Endemic Disease Control, Haizhu District Center for Disease Control and Prevention, Guangzhou, China; ^3^ Department of Medical Oncology, State Key Laboratory of Oncology in South China, Collaborative Innovation Center for Cancer Medicine, Sun Yat-sen University Cancer Center, Guangzhou, China; ^4^ Department of Ultrasound, The First Affiliated Hospital of Sun Yat-sen University, Guangzhou, China; ^5^ Department of Pathology and Laboratory Medicine, Perelman School of Medicine, University of Pennsylvania, Philadelphia, PA, United States; ^6^ Zhongshan School of Medicine, Sun Yat-sen University, Guangzhou, China; ^7^ Department of Breast Cancer, Cancer Center, Guangdong Provincial People’s Hospital, Guangdong Academy of Medical Sciences, Guangzhou, China

**Keywords:** DNA methylation, IRMGs, immune infiltration, prognosis, breast cancer

## Abstract

The role of DNA methylation of breast cancer-infiltrating immune cells has not been fully explored. We conducted a cohort-based retrospective study analyzing the genome-wide immune-related DNA methylation of 1057 breast cancer patients from the TCGA cohort and GSE72308 cohort. Based on patients’ overall survival (OS), a prognostic risk score system using 18 immune-related methylation genes (IRMGs) was established and further validated in an independent cohort. Kaplan–Meier analysis showed a clear separation of OS between the low- and high-risk groups. Patients in the low-risk group had a higher immune score and stromal score compared with the high-risk group. Moreover, the characteristics based on 18-IRMGs signature were related to the tumor immune microenvironment and affected the abundance of tumor-infiltrating immune cells. Consistently, the 18-IRMGs signatures showed similar influences on immune modulation and survival in another external validation cohort (GSE72308). In conclusion, the proposed 18-IRMGs signature could be a potential marker for breast cancer prognostication.

## Introduction

The biological behavior and clinical outcome of breast cancer are highly heterogeneous (1, 2). The molecular properties of breast cancer cells have been extensively studied to identify subgroups of patients having different treatment responses and prognosis for targeted therapy based on biomarkers (3–5). However, the tumor microenvironment is a complex mixture of malignant and non-malignant cells including immune cells which can affect the behavior and clinical outcomes of breast cancer. Up to date, the classification of the tumor microenvironment and its impact on prognosis are still poorly understood.

Immune cells from the microenvironment of breast cancer play an important role in determining tumor progression. Single-cell RNA sequencing of breast cancer has confirmed that there is a complex mixture of immune T cell subtypes in tumors (6, 7). Besides, a large number of transcriptomics analyses have been used to explore the immune microenvironment of breast cancer, which indicated that patients with different expression of genes involving multiple immune cells had different survival rates (8–11). Most of the previous research methods to decipher the characteristics of tumor immune microenvironment infiltration were based on the transcriptome (8, 9). DNA methylation, RNA and protein levels can be used as prognostic markers (12). But these markers have their own advantages and disadvantages. For example, DNA methylation and RNA sequencing results can be obtained by high-throughput chip or sequencing (13), which is more efficient and economical. However, high-throughput protein detection is time-consuming and expensive, and has not been widely used. However, few studies have explored the impact of immune cell infiltration on cancer from the perspective of DNA methylation patterns.

DNA methylation has established its role as the main epigenetic driving force in cancer progression and development (14–19). However, its contribution to defining the characteristics of the tumor microenvironment is still poorly understood. It has recently been shown that DNA hypomethylation promotes immune escape in corresponding tumors (15, 20, 21). Furthermore, DNA methylation patterns that predict the response of non-small cell lung cancer to immune checkpoint blockade (ICB) treatment have been revealed (22). DNA methylation patterns are also closely related to cell lineage and high levels of DNA methylation are often detected in blood and skin lineage. Finally, DNA methylation is associated with cellular and cell-free DNA derived from peripheral blood cells (23–25), and has been introduced as a complementary method for classifying the central nervous system (CNS) tumors (26). However, DNA methylation has not been widely used to determine the immune environment that occurs in the microenvironment of breast cancer.

Here, we identified DNA methylation markers, establishing an 18 immune-related methylation genes (IRMGs) signature, which could reflect multiple tumor-related immune cell subpopulations and divided the tumors into two clusters with different clinical and molecular characteristics, which were then validated in an independent dataset. This proposed signature could effectively predict the immune activity of the breast cancer microenvironment and the clinical prognosis of the patients.

## Materials and Methods

### Study Population

**Figure 1:**
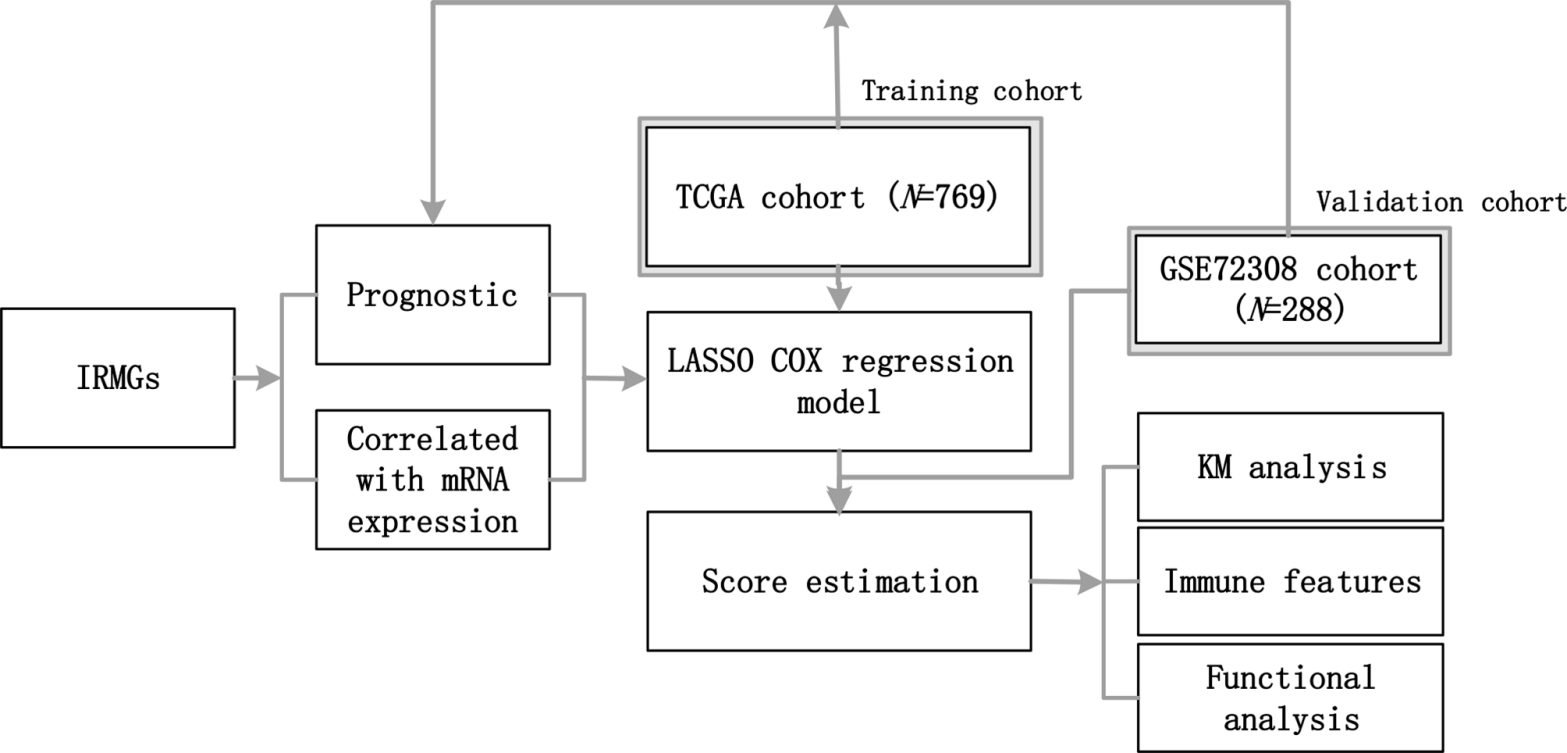
The workflow of this study. A group of IRMGs related to the prognosis of breast cancer was identified in the TCGA and GSE72308 data cohort. Using the LASSO Cox regression model, a new immune methylation cluster was constructed in the TCGA cohort, and its key role in breast cancer immune status and the prognosis was validated in the GSE72308 cohort. IRMGs, immune-related methylation genes; TCGA, The Cancer Genome Atlas; GEO, Gene Expression Omnibus; LASSO, least absolute shrinkage and selection operator; KM, Kaplan-Meier.

Breast cancer datasets from The Cancer Genome Atlas (TCGA) and Gene Expression Omnibus (GEO) were downloaded and the workflow is illustrated in **Figure 1**. GSE72308contained a set of data obtained from methylation array analysis, which has been used to evaluate the characteristics of immune response based on DNA methylation in breast cancer and other cancers (27). Only patients who met the following criteria were selected: (1) confirmed pathological diagnosis of invasive breast cancer; (2) available DNA methylation and overall survival (OS) data. Patients without active follow-up and transcriptomic data in the TCGA were excluded. In this study, molecular subtypes classified based on immunohistochemical detection in TCGA and GSE72308 were used for analysis. The number of patients in different molecular subtypes in TCGA are: Basal (n=193), HER (n=282), LumA (n=581), LumB (n=219), Normal (n=143).The number of patients in different molecular subtypes in GSE72308 are: Basal (n=65), HER2 (n=56), LumA (n=52), LumB (n=63).This study was based on the analysis of the TCGA and GSE72308 cohort, and was therefore deemed exempt from institutional review board approval by The Sun Yat-sen University Cancer Center, and informed consent was waived. We conducted this study in accordance with the ethical standards of the World Medical Association Declaration of Helsinki.

### Data Acquisition and Generation of Immune Methylation Profiles

First, we downloaded a list of immune-related genes (**Supplementary List 1**) from the Immunology Database and Analysis Portal (ImmPort, https://www.immport.org). A total of 1826 immune-related genes for subsequent analysis. Subsequently, we downloaded the methylation data of breast cancer patients from the TCGA and GSE72308 databases. The TCGA methylation profile was obtained from the Xena database (https://xenabrowser.net/). The GSE72308 methylation profile was obtained from the GEO database (https://www.ncbi.nlm.nih.gov/geo/). Finally, the DNA methylation sites of the aforementioned immune-related genes (1812 genes downloaded from ImmPort) were screened in the TCGA and GSE72308 methylation profile. The DNA methylation sites of these immune-related genes are defined as IRMGs.

Besides, the expression profiles of the TCGA cohort were downloaded from the TCGA data portal (https://portal.gdc.cancer.gov/repository). Then we extracted the DNA methylation quantitative index β values [βvalue=methylation signal/(methylation signal + non-methylation signal)]of these genes and the corresponding RNA expression profiles. Subsequently, the correlation between DNA methylation level and RNA expression level was analyzed one by one.

The ensemble IDs were mapped to gene symbols according to the annotation of Homo_sapiens.GRCh38.91.chr.gtf from the ENSEMBLE website. The “limma” package in R was used for gene expression normalization using the scale method (28). The average RNA expression was calculated for duplicates, and genes with low abundance were discarded.

### Development of the DNA Methylation-Based Immune Profiling

The Univariate Cox regression was used to determine the immune-related methylation genes (IRMGs) associated with OS. The Least Absolute Shrinkage and Selection Operator (LASSO) Cox regression model was further applied to determine the key features and corresponding coefficients for the model construction (29). The LASSO Cox regression was performed using the “glmnet” package of the R software, and the ideal coefficient was estimated based on the partial likelihood deviation with ten-fold cross-validation (30). The optimal log λ was -4.37. To quantify the comprehensive impact of immune methylation status, a new score was calculated based on the features selected by the LASSO model.

First, we obtained IRMGs significantly related to the prognosis of TCGA and GSE72308 cohorts through the univariate cox regression analysis of IRMGs on overall survival. Then, IRMGs with a proper correlation between expression and DNA methylation level (*r*>0.2 and *P*<0.05) were selected and added to the LASSO cox regression model for modeling to calculate the score and standardization through the obtained coefficients using the following formula:


$$\text{Sum}=\sum_{i=1}^{\text{n}}\left(\text{IMRG}\times \text{Coe}{\text{f}}_{\text{i}}\right)\text{Score}=(\text{Sum}-\text{Min})/\text{Max}$$

### Tumor Microenvironment and Function Analysis

The Cell-type Identification By Estimating Relative Subsets Of RNA Transcripts (CIBERSORT) analysis was used to identify immune cell types. The expression matrix was uploaded using the online analysis platform (https://cibersort.stanford.edu), and the proportion of infiltrating immune cells was estimated by the LM22 signature with 1000 permutations (31). Subsequently, the criterion of *P*<0.05 was used to select qualified samples. The xCell analysis was performed following its guidelines (https://xcell.ucsf.edu) (32). Immune and stromal scores were further estimated *via* the Estimation of Stromal and Immune cells in MAlignant Tumor tissues using Expression data (ESTIMATE) algorithm with the “estimate” package in R to quantify the immune and stromal components (33). The MCP-counter scores of immune-related active cells and fibroblasts were evaluated using the “MCPcounter” package in R (34). The cluster Profiler package of the R software was used for GO analysis (35). According to previous research, the “fGSEA” software package in R (version 4.0.1) was used to perform GSEA analysis to explore pathway enrichment between the low- and high-risk groups (36).

### Statistical Analysis

The Univariate Cox regression was used to identify prognostically relevant IRMGs with a cutoff value of *P*<0.05. Crucial signatures involved in immune-related methylation clusters were identified using the LASSO Cox regression model. The optimal cut-off value for survival analysis was determined using the “survminer” package in R, and the OS of different subgroups were compared using the Kaplan-Meier method with the log-rank test. For the Kaplan-Meier analysis, the cut-off values for the score-high and score-low groups were based on the median score. The follow-up time was 6 years. Time-dependent receiver operator characteristic (ROC) analyses were performed using the “timeROC” package in R (34). Spearman’s correlation test was used for Score-related analysis. All statistical analyses were performed using the R software (Version 4.0.1). A *P* value of <0.05 was considered statistically significant, and all *P* values were two-tailed.

## Results

### DNA Methylation-Based Immune Profiling of Breast Cancer

**Figure 2:**
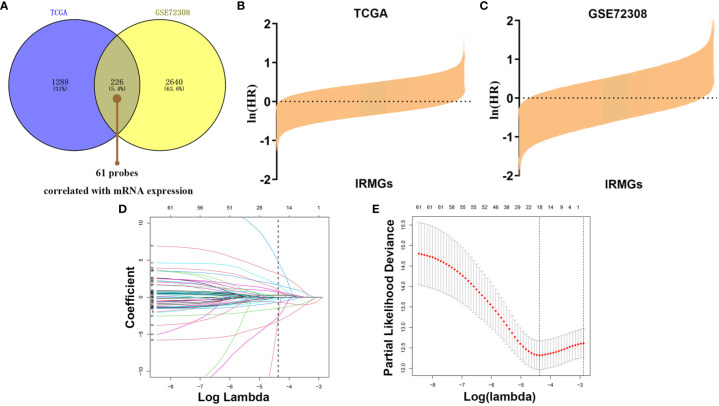
Identification of prognostic IRMGs in breast cancer. **(A)** Venn plot shows that the 61 IRMGs identified in the two cohorts were associated with mRNA expression. **(B, C)** Bar graph showing the hazard ratio of IRMG in the TCGA cohort and GSE72308 cohort. The bars represent *95%CI*. The Univariate Cox regression was used for data analysis. **(D, E)** The LASSO Cox regression model was constructed from the 61 signature IRMGs, and the adjustment parameter (λ) was calculated based on the partial likelihood deviation with ten-fold cross-validation. The optimal log λ value is -4.37, as shown by the vertical black line in the curve. According to the best fit contour, an 18-IRMGs signature was determined. IRMGs, immune-related methylation genes; TCGA, The Cancer Genome Atlas; GEO, Gene Expression Omnibus; LASSO, least absolute shrinkage and selection operator.

To explore the pattern of immune infiltration based on DNA methylation in breast cancer, we first compiled the TCGA methylation profile and GSE72308 methylation profile (**Figure 1**) to identify the corresponding IRMGs. A population of 769 and 288 patients from the TCGA and GSE72308 were included in this study. The IRMGs data from the TCGA and GSE72308 were subjected to univariate Cox proportional hazard regression analysis, of which a total of 226 IRMGs (**Supplementary List 2**) were found significantly related to the OS of breast cancer patients (P<0.05) in both cohorts and were identified as candidate markers (**Figures 2A–C**). Subsequently, 61 IRMGs (**Supplementary List 3**) were significantly correlated with corresponding mRNA expression (|r|>0.2, P<0.05) and were selected for the prognosis prediction model. Based on these, the LASSO Cox regression model was used to construct a prognostic model for the OS stratification of the patients from the TCGA data set (N=769). First, we determined the penalty value [log(lambda)=-4.37] according to the lowest point of the **Figure 2E** curve, and drew a vertical line at the position of log(Lambda)=-4.37 in **Figure 2D**. Each curve in **Figure 2D** represented a variable, and the curve that intersected with vertical line at the position of log(Lambda)=-4.37 was the final included in the model. The ordinate corresponding to the variable was the regression coefficient of the variable. In the regression equation, the regression coefficient represented the contribution of the variable.

**Figure 3:**
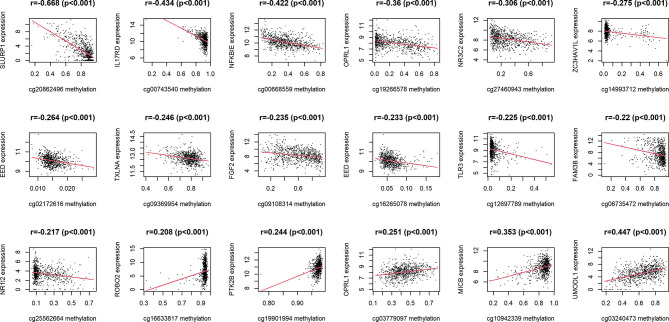
The correlation between IRMGs identified in the LASSO model using the TCGA cohort and their corresponding mRNA expression levels.

18 IRMGs were selected according to the method of partial likelihood deviance, and the corresponding coefficients were generated with the best logλ of -4.37. **Supplementary List 4** shows the positions of these 18 IRMGs in the corresponding genes. The hazard ratio model consisting of 18 methylation sites(cg06735472, cg20862496, cg02172616, cg09108314, cg09369954, cg03779097, cg27460943, cg16633817, cg19901994, cg16265078, cg10942339, cg03240473, cg19266578, cg00668559, cg12697789, cg14993712, cg25562664, cg00743540) was selected as the best prognostic model for predicting OS (**Figures 2D, E**). The genes corresponding to these 18 methylation sites were SLURP1(cg240862496), IL17RD(cg00743540), NFKBIE(cg00668559), OPRL1(cg19266578), NR3C2(cg27460943), ZC3HAV1L(cg14993712), EED(cg02172616), TXLNA(cg09369954), FGF2(cg09108314), EED(cg16265078), TLR3(cg12697789), FAM3B(cg06735472), NR1I2(cg25562664), ROBO2(cg16633817), PTK2B(cg19901994), OPRL1(cg03779097), MICB (cg10942339)and UMODL1(cg03240473). **Figure 3** shows the correlation coefficient and *P* value between the IRMGs selected by the LASSO model and their corresponding mRNA expression.

**Figure 4:**
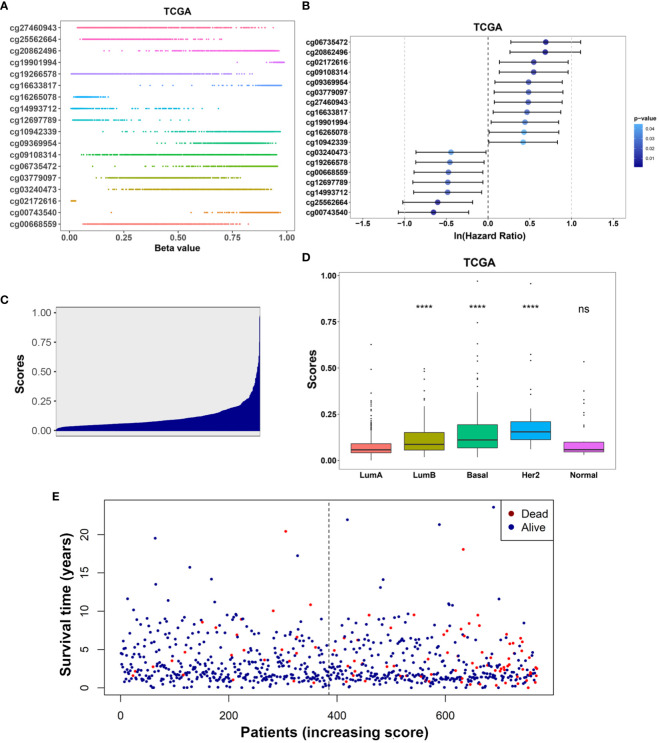
**(A)** Distribution of methylation levels of the 18-IRMGs signature in the TCGA cohort; **(B)** Multivariate Cox regression analysis results of the 18-IRMGs signature corresponding to OS in the TCGA cohort. **(C)** Distribution of risk scores in the TCGA cohort; **(D)** Comparison of risk scores of different molecular subtypes in the TCGA cohort (****: p<=0.0001, ns: p>0.05); **(E)** The distribution of patients in different risk scores according to survival status and survival time. IRMGs, immune-related methylation genes; OS, overall survival; TCGA, The Cancer Genome Atlas; Her2, human epidermal growth factor receptor 2.

Kaplan-Meier analysis further confirmed the prognostic value of each of these 18 IRMGs (**Supplemental Figure 1**). Patients with higher methylation levels of cg03240473, cg19266578, cg00668559, cg12697789, cg14993712, cg25562664, and cg00743540 had poorer prognosis, while patients with higher methylation levels of cg06735472, cg20862496, cg02172616, cg09108314, cg09369954, cg03779097, cg27460943, cg16633817, cg19901994, cg16265078, cg10942339 had better prognosis; which was consistent with the results of the Lasso Cox regression analysis. The methylation values of the 18 IRMGs in TCGA and the corresponding *HR* and *95%CI* are shown in **Figures 4A, B**. Among the 18 IRMGs, 11 IRMGs was associated with improved prognosis while 7 with poor prognosis.

The risk score of the TCGA training cohort was calculated using the coefficients obtained by the above-mentioned LASSO algorithm. The risk score distribution of the 18 immune methylation markers in the TCGA training cohort is shown in **Figure 4C**. The risk scores of different molecular subtypes were significantly different, with HER2 positive scores being the highest, followed by the basal-like subtype and luminal B subtype. The risk score of these three subtypes was significantly higher than that of luminal A (**Figure 4D**). However, there was no significant correlation between the risk score and tumor size, lymph node metastasis, TNM stage, and age. The distribution of risk scores based on different survival time and survival status (alive or dead) is shown in **Figure 4E**. From this, we can observe that the patients who died (red dots) are more distributed in the high-risk group. Second, patients in the low-risk group had a longer survival time.

### The 18-IRMGs Signature Was Significantly Associated With Molecular Characteristics and Immune Features

**Figure 5:**
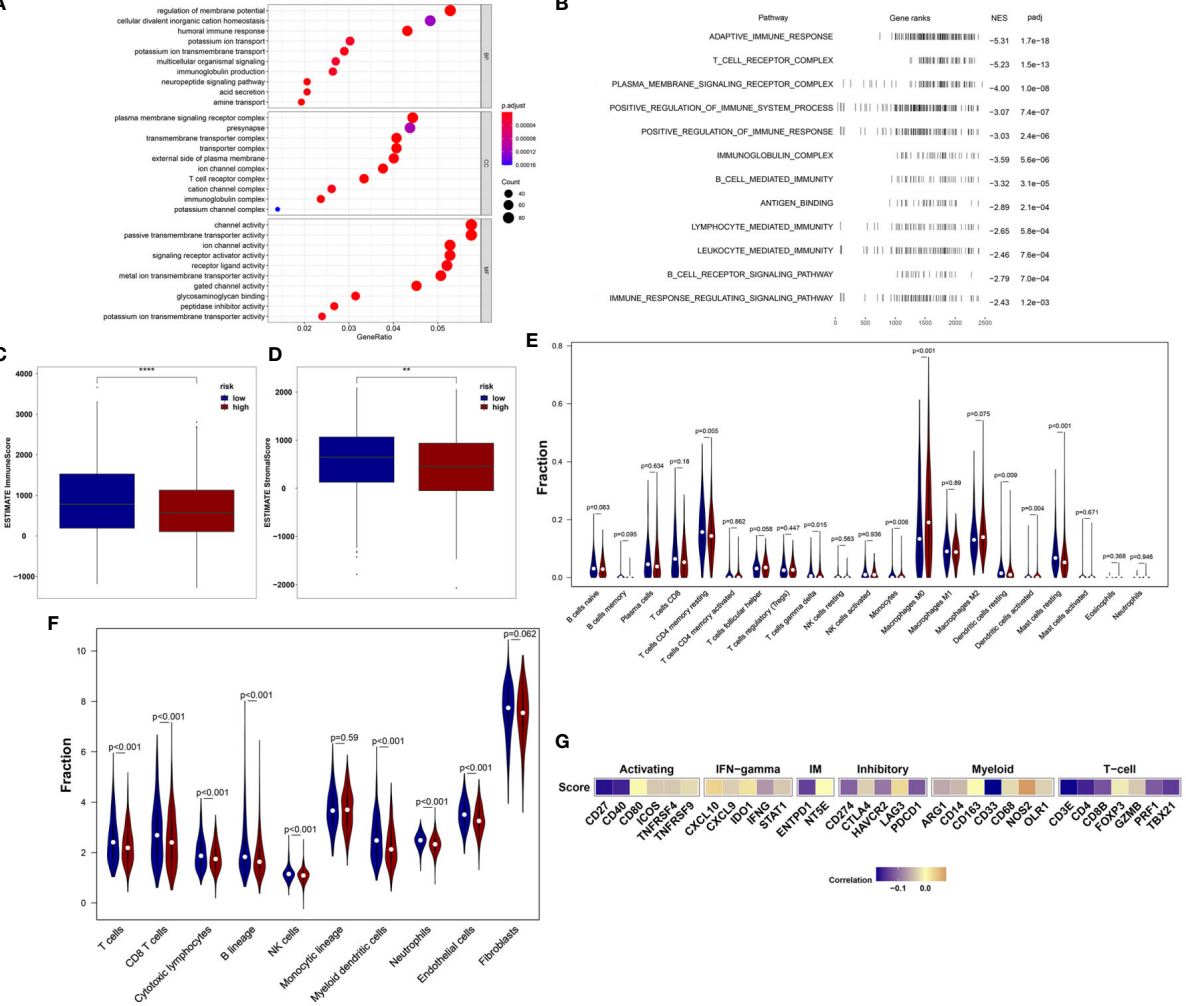
18-IRMGs signature related immune characteristics and immune cells. **(A)** GO analysis of the group based on18-IRMGs signature. **(B)** GSEA is related to immune-related signals based on the 18-IRMGs signature. **(C, D)** ESTIMATE immune score and stromal score between groups based on IMI (**: p<=0.01, ****: p<=0.0001). **(E)** Comparison of infiltrating immune cells (CIBERSORT) between the two risk groups. **(F)** Comparison of infiltrating immune cells (MCP counter) between the two risk groups. **(G)** Correlation between risk scores and immune-related genes in the TCGA cohort. IRMGs, immune-related methylation genes; GO, Geneontology; GSEA, Gene Set Enrichment Analysis; ESTIMATE, Estimation of Stromal and Immune cells in MAlignant Tumor tissues using Expression data; CIBERSORT, Cell-type Identification By Estimating Relative Subsets Of RNA Transcripts.

We first analyzed the enriched pathways of the 18-IRMGs signature through biological function enrichment analysis. The results showed that genes were significantly enriched in immune-related pathways of GO categories (**Figure 5A**), including humoral immune response, immunoglobulin production, T cell receptor complex, and immunoglobulin complex. Furthermore, Gene Set Enrichment Analysis (GSEA) analysis revealed 12 important pathways related to the 18-IRMGs signature, including adaptive immune response, T cell receptor complex, immunoglobulin complex, antigen binding, B cell receptor signaling pathway, lymphocyte-mediated immunity, neutrophil-mediated immunity, and more (**Figure 5B**). To study the effect of the 18-IRMGs signature on the immune microenvironment of breast cancer, we evaluated the immune score and stromal score between the high- and low-risk groups. The results showed a significant difference in immune score and stromal score between the high-risk and low-risk groups (**Figures 5C, D**). The immune score and stromal score of the low-risk group were significantly higher than that of the high-risk group (*P*<0.05).

Further, the ratio of 22 immune cell types between the two subgroups was analyzed. We first used the CIBERSORT algorithm to estimate the proportion of immune cells in the TCGA cohort (**Figure 5E**), and found that the low-risk group had a higher percentage of anti-tumor immune cells, including gamma delta (γδ) T cells (*P*<0.05), CD4+ memory T cells (*P*<0.01), mast cells (*P*<0.01), and resting dendritic cells (*P*<0.01). In addition, patients in the high-risk group showed a higher proportion of immunosuppressive cells, such as M0 macrophages. Although we observed that the level of activated dendritic cells in the high-risk group was higher than that in the low-risk group, the absolute level in both groups was very low, even far lower than other types of immune cells. The effect of such a low level of activated dendritic cells may be almost negligible. Then, the MCP-counter algorithm was used to estimate the proportion of immune cells in the TCGA cohort (**Figure 5F**). Consistent with the above results, patients in the low-risk group also demonstrated a higher percentage of anti-tumor immune cells, including T cells, CD8+ T cells, cytotoxic T cells, B lineage, myeloid dendritic cells, and neutrophils. In addition, the risk score was negatively correlated with the mRNA expression of immune checkpoints CD27, CD40, ENTPD1, PDCD1, CD274, HAVCR2, CD33, CD4, TBX21, CD8B, and PRF1, but positively correlated with the expression of NOS2 (**Figure 5G**). We also observed that the risk score was mainly negatively correlated with the expression of immune checkpoints related to T cells.

### Prognostic Value of the 18-IRMGs Signature

The development of convenient tools for early diagnosis and treatment guidance of diseases remains a critical clinical issue. To further clarify the prognostic and prognostic value of the 18-IRMGs signature in breast cancer, ROC analysis, and the Kaplan-Meier method were used to assess the prognosis in the TCGA cohort.

**Figure 6:**
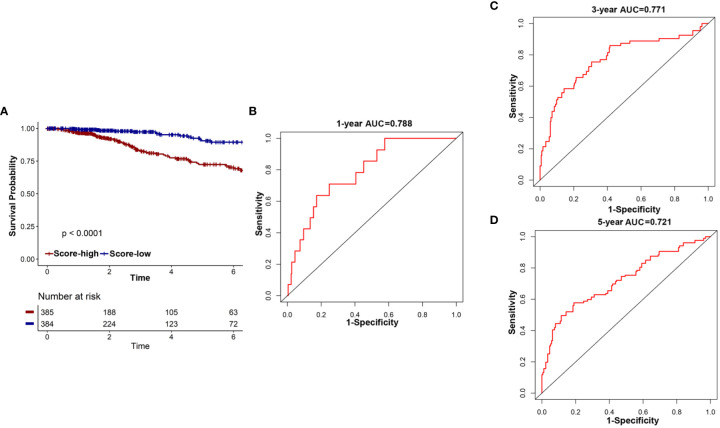
The potential indicator value of 18-IRMGs signature in the prognosis of TCGA breast cancer. **(A)** Kaplan-Meier plot of OS between the two risk groups in the TCGA cohort. The log-rank test was used for data analysis. **(B–D)** Time-dependent ROC analysis (1, 3, and 5 years) based on 18-IRMGs signature in breast cancer patients in the TCGA cohort. IRMGs, immune-related methylation genes; OS, overall survival; ROC, receiver operating characteristic; TCGA, The Cancer Genome Atlas; AUC, area under the curve.

First, the overall survival of patients with different risk scores was compared. In the TCGA cohort, patients in the low-risk group had a better OS than those in the high-risk group (**Figure 6A**). A time-related ROC analysis was performed and the area under the curve (AUC) was calculated at different time points based on the availability of data (**Figures 6B–D**). The results suggested that the corresponding AUCs of the ROC analysis for 1-, 3-, and 5-year follow-up in the TCGA cohort were 0.788, 0.771, and 0.721, respectively (**Figures 6B–D**). This indicated that the 18-IRMGs signature had good prognostic value in both short-term and long-term follow-up.

### Validation of the 18-IRMGs Signature for Breast Cancer Survival Prediction in an Independent Cohort

**Figure 7:**
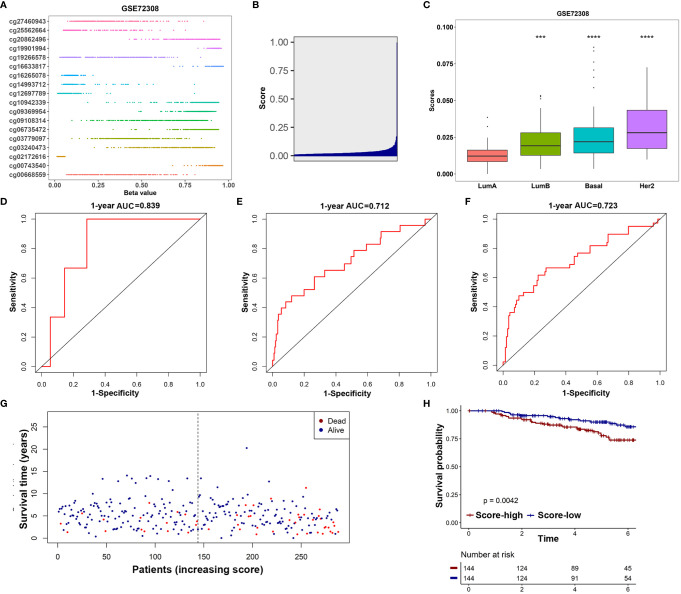
Validation of the prognostic value of 18-IRMGs signature for OS in the GSE72308 cohort. **(A)** Distribution of methylation levels of 18 IRMGs in the GSE72308 cohort; **(B)** Distribution of risk scores in the GSE72308 cohort; **(C)** Comparison of risk scores of different molecular subtypes in the GSE72308 cohort (***: p<=0.001, ****: p<=0.0001); **(D–F)** Time-dependent ROC analysis (1, 3, and 5 years) based on 18-IRMGs signature in breast cancer patients in the GSE72308 cohort; **(G)** The distribution of patients of different risk scores according to survival status and survival time; **(H)** Kaplan-Meier plot of OS between the two risk groups in the GSE72308 cohort. The log-rank test was used for data analysis. IRMGs, immune-related methylation genes; OS, overall survival; ROC, receiver operating characteristic; AUC, area under the curve; Her2, human epidermal growth factor receptor 2.

The GSE72308 was used as the independent external validation cohort (*N*=288). First, the methylation values of the 18 IRMGs and risk score distribution of all the patients in the GSE72308 cohort are shown in **Figures 7A, B**. Consistent with the TCGA cohort, in the GSE72308 cohort, it was also observed that the HER2 positive subtype had the highest risk score, followed by basal-like subtypes and luminal B subtypes. The risk scores of these three subtypes were significantly higher than the luminal A subtype (**Figure 7C**). Similarly, high- or low-risk patients were grouped according to the median risk score. The results showed that the 18-IRMGs signature performed well, and compared with the high-risk group, the OS of patients in the low-risk group was significantly longer (*P*<0.05) (**Figure 7H**). The ROC curve over time shows that the 18-IRMGs signature had good accuracy, with 0.839 for 1 year-AUC, 0.712 for 3 years-AUC, and 0.723 for 5 years-AUC (**Figures 7D–F**). The distribution of risk scores of breast cancer patients from the GSE72308 cohort based on different survival time and survival status (alive or dead) was also shown in **Figure 7G**.

## Discussion

In this study, we provided new insights into the heterogeneity of the tumor immune microenvironment of breast cancer and confirmed that the specific characteristics of immune methylation have an important prognostic value. We analyzed the relationship between DNA methylation and tumor immunity of breast cancer through the IRGMs set, and identified two immune methylation clusters significantly related to patient survival, which was then validated in an independent cohort. These demonstrated the relationship between immune methylation characteristics and corresponding immune cell infiltration in the tumor microenvironment and patient prognosis.

Some recent studies have suggested that there are abnormal methylation events in breast tumors, and specific DNA methylation patterns may be closely related to breast cancer immune microenvironment, molecular subtypes, and recurrence. Dedeurwaerder et al. (37) conducted DNA methylation analysis on 248 breast tissues and found that DNA methylation analysis can reflect the cell type composition of the tumor microenvironment, especially the T lymphocyte infiltration of the tumor. What they found also strongly proved that DNA methylation can indeed help better understand the complex relationship between tumor cells and the immune microenvironment. Holm et al. (38) used an array-based methylation assay to analyze the methylation status of 807 cancer-related genes in 189 fresh frozen primary breast tumors and 4 normal breast tissue samples. They found that basal-like, luminal A and luminal B subtypes of breast cancer have specific methylation characteristics, suggesting that methylation may play an important role in the development of breast cancer. Kamalakaran et al. (39) found that the DNA methylation pattern in luminal breast cancer is different from non-luminal subtypes, and the DNA methylation pattern can be independent of other clinical variables to identify the risk of recurrence.

Based on the TCGA data set of 769 breast cancer samples that met the inclusion criteria, the current study has identified prognostic immune methylation features with potential clinical applicability. The risk score obtained from the 18-IRMGs signature effectively divided breast cancer patients into high-risk and low-risk groups. In the TCGA cohort, the OS of the high-risk group was shorter than that of the low-risk group (p<0.001) and also demonstrated good prognostic performance (AUC of 1, 3, and 5 years are 0.788, 0.771, and 0.721, respectively). Besides, the results of the univariate Cox regression (**Figure 4B**) and Kaplan-Meier (**Supplementary Figure 1**) for 18 individual immune methylation sites showed that each immune methylation site could also distinguish high-risk and low-risk patients. This indicated that a single immune methylation site may play a role in prognostic prediction, and the combination of 18 methylation sites provided better prognostication ability. Based on our existing knowledge, the prognostic value of related multiple immune methylation signatures in breast cancer has not been reported. Therefore, this study provides new insights on the combination of epigenetic biomarkers helping to improve the risk stratification and survival prediction of breast cancer patients.

Considering that ideal prognostic markers can also effectively stratify risk in other independent cohorts, we used the GSE72308 cohort to further evaluate the practicality of the 18-IRMGs signature. It performed well in distinguishing the low-risk and high-risk groups of the GEO cohort, and the prediction accuracy of the GSE72308 cohort was consistent with the TCGA cohort (1-year-AUC=0.839, 3-years-AUC =0.712, and 5-years-AUC=0.723).

The blockade of immune checkpoints such as PD-1, PD-L1, and CTLA-4 has shown impressive results in a series of solid cancers (especially melanoma and non-small cell lung cancer). Currently, there are many ongoing and planned trials of these drugs in breast cancer. However, only a small percentage of breast cancer patients respond to immune checkpoint blockade(ICB) treatment, and the identification of ICB response biomarkers and drug resistance modifiers is a key challenge. DNA methylation plays a vital role in cell lineage regulation and can be used as a specific molecular marker for immune response measurement. The role of DNA methylation in the immune response to cancer is becoming increasingly important and it is currently considered to be closely related to the efficacy of immunotherapy for melanoma and other tumors. Recently, Duruisseaux M et al. (22) found that a microarray DNA methylation signature could predict the efficacy of anti-PD-1 therapy in stage IV NSCLC patients. Similarly, Kim et al. also found that methylation patterns could predict the clinical benefit of immunotherapy in lung cancer (40). In the present study, our proposed 18-IRMGs signature was found to be significantly related to the prognosis of breast cancer patients.

Further analyses showed that the characteristics based on 18-IRMGs signature were related to the tumor immune microenvironment and affected the abundance of tumor-infiltrating immune cells. The stromal cell score and the immune score of the high-risk group were significantly lower than the low-risk group. Further analysis showed that in the low-risk group, the infiltration level of quiescent mast cells, CD4 memory T cells, mast cells, gamma delta T cells and resting dendritic cells was higher than the high-risk group. In contrast, the infiltration level of macrophages M0 cells in the high-risk group were higher than those in the low-risk group. Although we observed that the activated dendritic cells in the high-risk group was higher than that in the low-risk group, the absolute level in both groups was very low, even far lower than other types of immune cells. The effect of such a low level of activated dendritic cells may be almost negligible. In general, the significant difference in survival rate between the two groups may be related to the difference in the immune microenvironment of the two groups. This finding is consistent with the results of previous studies, which showed that patients with low immune scores had a worse survival than patients with high immune scores.

Undeniably, our research had several limitations. First, despite the identification and validation of the 18-IRMGs signature, additional prospective external verification is required in a multicenter cohort to confirm the study findings. Second, it is necessary to further study the regulatory mechanism of DNA methylation in tumor immune microenvironment(TIME) to reshape TIME and improve precision immunotherapy for breast cancer. Third, there is no data on DNA methylation in breast cancer patients receiving immunotherapy, so it is unclear whether they could also be used as a marker for predicting ICB efficacy. Fourth, this study mainly used two independent databases (GSE72308 and TCGA data sets) to analyze the relationship between IRMG and immune activity. The results of this study have not been verified by extensive *in vitro* experiments. Fifth, we did not find any significant correlation between TNM staging and the 18-IRMGs signature, suggesting that the two are independent of each other in judging prognosis.

## Conclusion

In summary, we identified and validated an 18-IRMGs signature that was significantly associated with OS in independent cohorts. The proposed 18-IRMGs signature demonstrated promising accuracy in stratifying breast cancer patients based on their survival differences and could be used as a guide to assess the need for adjuvant therapy. In addition, the 18-IRMGs signature was closely related to the tumor immune microenvironment and may be used to select patients who respond to immunotherapy.

## Data Availability Statement

The data used and analyzed during the current study are available from the corresponding author on reasonable request.

## Author Contributions

AY, YZ,YK, and XW conceived and designed the experiments. AY, YZ,YK, XW, FY, LZ, XZ, ML, SL, XA, and WX performed the experiments and analyzed the data. XA and WX wrote the paper. All authors contributed to the article and approved the submitted version.

## Funding

This research is funded by the National Natural Science Foundation of China (approval No.: 82003066), Guangzhou basic and applied basic research project (202102020168), and special science and Technology Fund (Doctoral entrepreneurship project) of Guangdong People’s Hospital and Guangdong Provincial Medical Research Fund (a2021080).

## Conflict of Interest

The authors declare that the research was conducted in the absence of any commercial or financial relationships that could be construed as a potential conflict of interest.

## Supplementary Material

Supplementary Figure 1 Kaplan-Meier plot of overall survival for 18 individual immune methylation sites showed that each immune methylation site could distinguish high-risk and low-risk patients in the TCGA cohort. The log-rank test was used for data analysis.

Supplementary List 1 List of 1812 immune-related genes from ImmPort.

Supplementary List 2 A total of 226 IRMGs were found significantly related to the OS of breast cancer patients.

Supplementary List 3 The 61 IRMGs were used in prognostic prediction models.

Supplementary List 4 The positions of the 18 IRMGs in the corresponding genes.

## Abbreviations

IRMGs, immune-related methylation genes; OS, overall survival; ROC, receiver operating characteristic; ICB, immune checkpoint blockade; CNS, central nervous system (CNS); TCGA, The Cancer Genome Atlas; GEO, Gene Expression Omnibus; IRGs, immune-related genes; LASSO, least absolute shrinkage and selection operator; CIBERSORT, Cell-type Identification By Estimating Relative Subsets Of RNA Transcripts; AUC, area under the curve; TIME, tumor immune microenvironment; GO, Geneontology; GSEA, Gene Set Enrichment Analysis; Her2, human epidermal growth factor receptor 2.
