# Corrigendum: Identification and validation of immune-related methylation clusters for predicting immune activity and prognosis in breast cancer

**DOI:** 10.3389/fimmu.2024.1372991

**Published:** 2024-01-31

**Authors:** Anli Yang, Ying Zhou, Yanan Kong, Xiaoli Wei, Feng Ye, Lijuan Zhang, Xian Zhong, Mingyue Li, Shilin Lu, Xin An, Weikai Xiao

**Affiliations:** ^1^ Department of Breast Oncology, State Key Laboratory of Oncology in South China, Collaborative Innovation Center for Cancer Medicine, Sun Yat-sen University Cancer Center, Guangzhou, China; ^2^ Department of Infectious Diseases and Endemic Disease Control, Haizhu District Center for Disease Control and Prevention, Guangzhou, China; ^3^ Department of Medical Oncology, State Key Laboratory of Oncology in South China, Collaborative Innovation Center for Cancer Medicine, Sun Yat-sen University Cancer Center, Guangzhou, China; ^4^ Department of Ultrasound, The First Affiliated Hospital of Sun Yat-sen University, Guangzhou, China; ^5^ Department of Pathology and Laboratory Medicine, Perelman School of Medicine, University of Pennsylvania, Philadelphia, PA, United States; ^6^ Zhongshan School of Medicine, Sun Yat-sen University, Guangzhou, China; ^7^ Department of Breast Cancer, Cancer Center, Guangdong Provincial People’s Hospital, Guangdong Academy of Medical Sciences, Guangzhou, China

**Keywords:** DNA methylation, IRMGs, immune infiltration, prognosis, breast cancer

In the published article, there was an error regarding the affiliations for Weikai Xiao. The affiliation of Weikai Xiao should only be 7, but not 1.

In the published article, there was also an error in the Funding statement. This previously stated: The approval number of the National Natural Science Foundation of China was displayed as “8203066”. And the approval number of Guangzhou basic and applied basic research project (approved in 2021).

The correct Funding statement appears below.

**FUNDING**

This research is funded by the National Natural Science Foundation of China (approval No.: 82003066), Guangzhou basic and applied basic research project (202102020168), and special science and Technology Fund (Doctoral entrepreneurship project) of Guangdong People’s Hospital and Guangdong Provincial Medical Research Fund (a2021080).

The authors apologize for these error and state that they do not change the scientific conclusions of the article in any way. The original article has been updated.

