# Supplementary figures and images for: Identification and Validation of Immune-Related Methylation Clusters for Predicting Immune Activity and Prognosis in Breast Cancer
Source: Front Immunol. 2021 Jun 30;12:704557. doi: 10.3389/fimmu.2021.704557 (PMC8278823; doi:10.3389/fimmu.2021.704557)

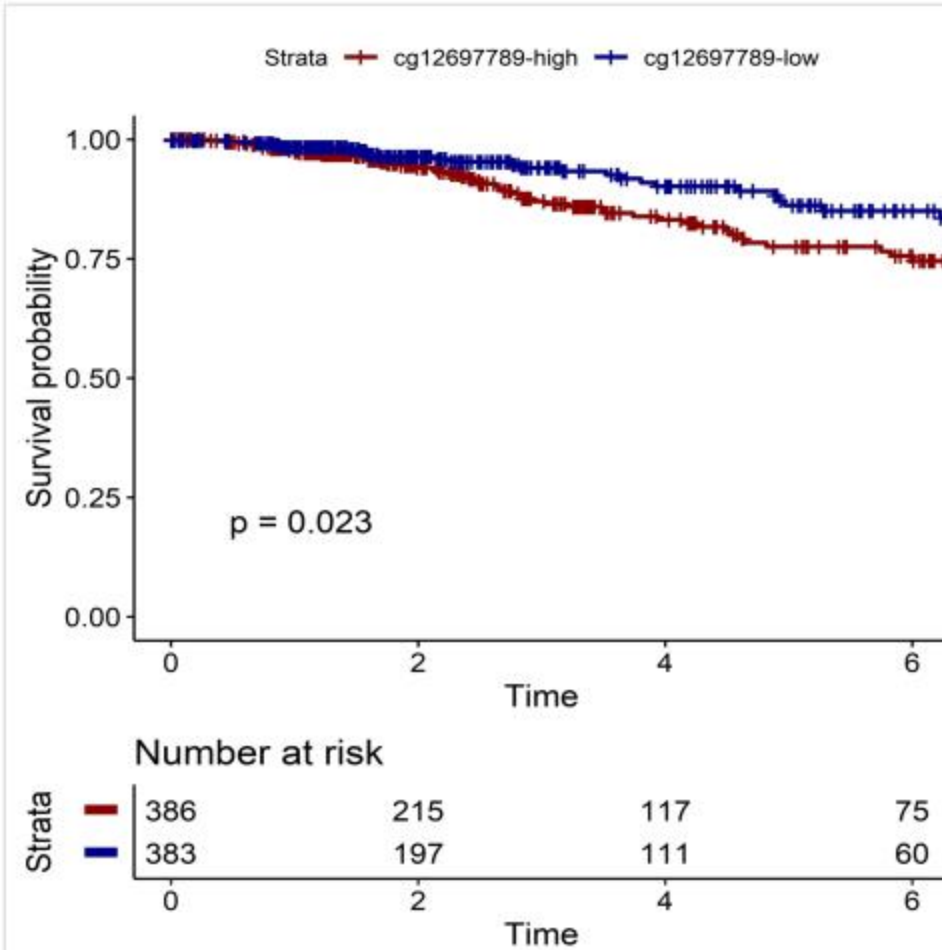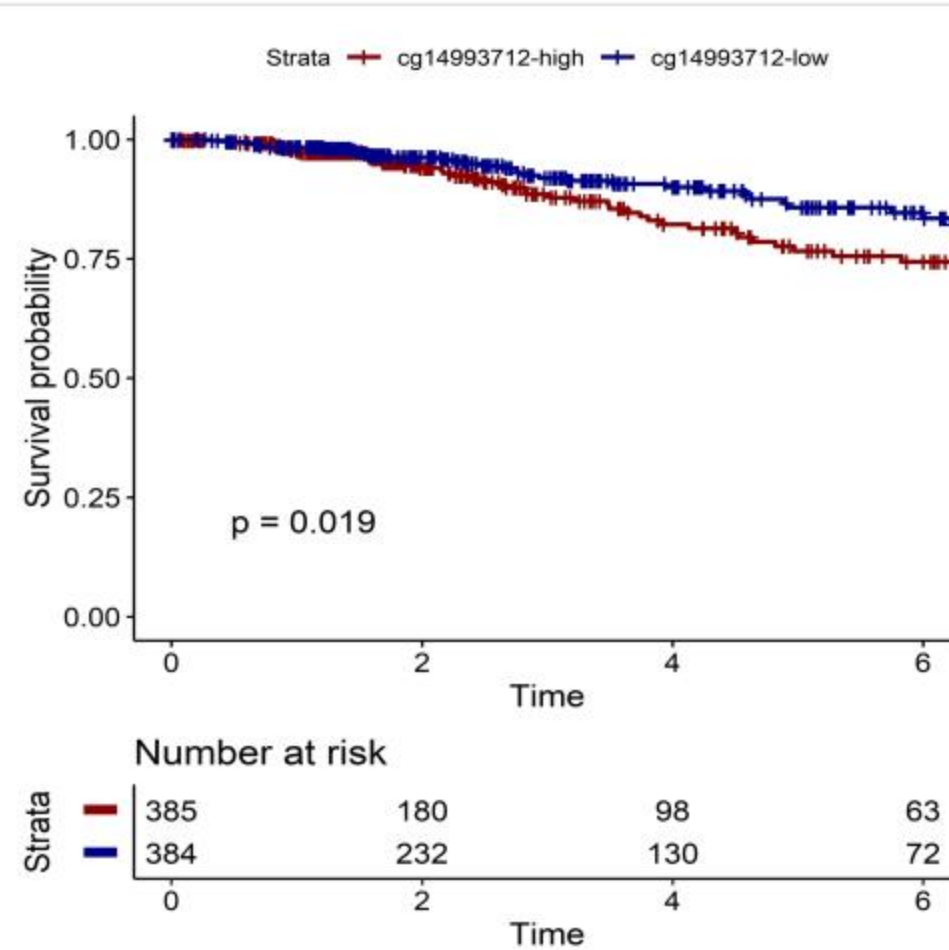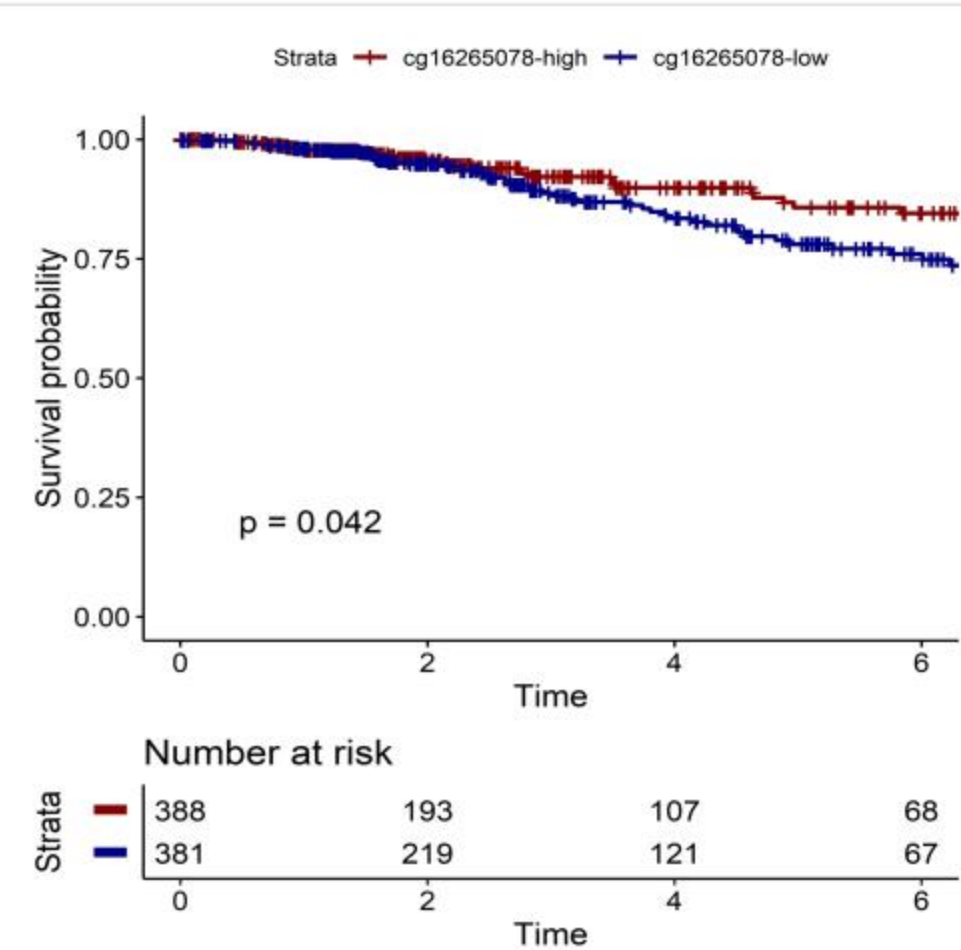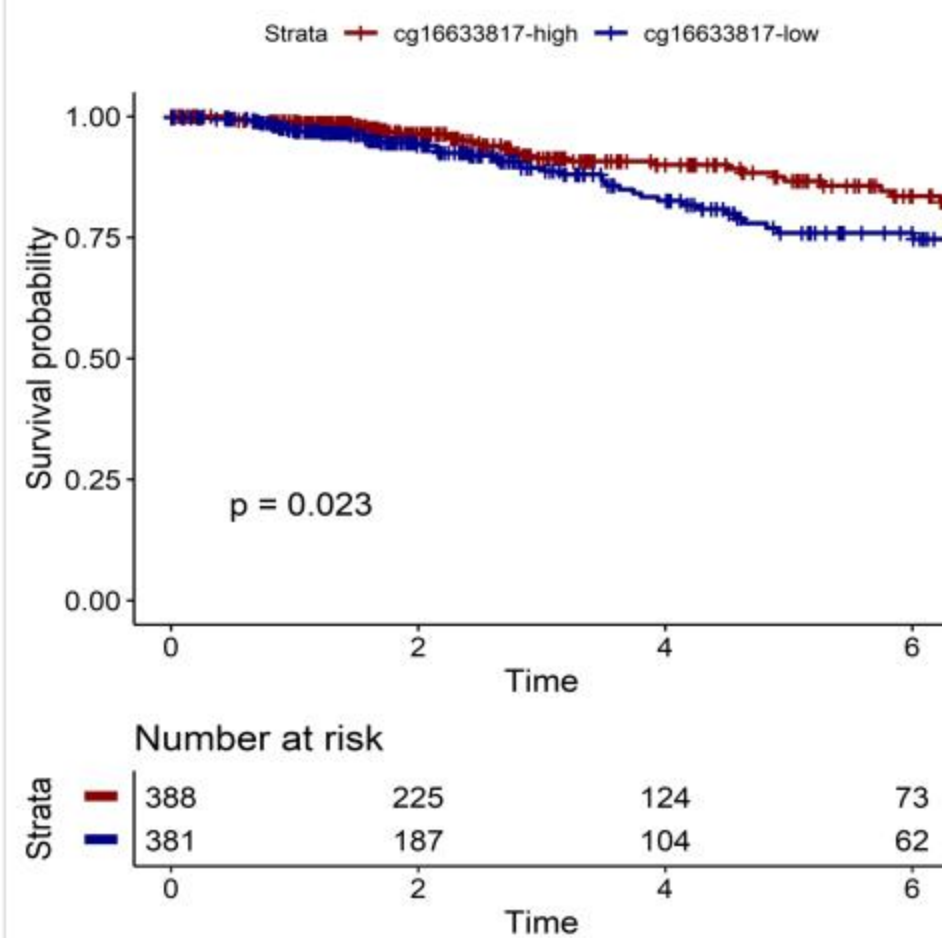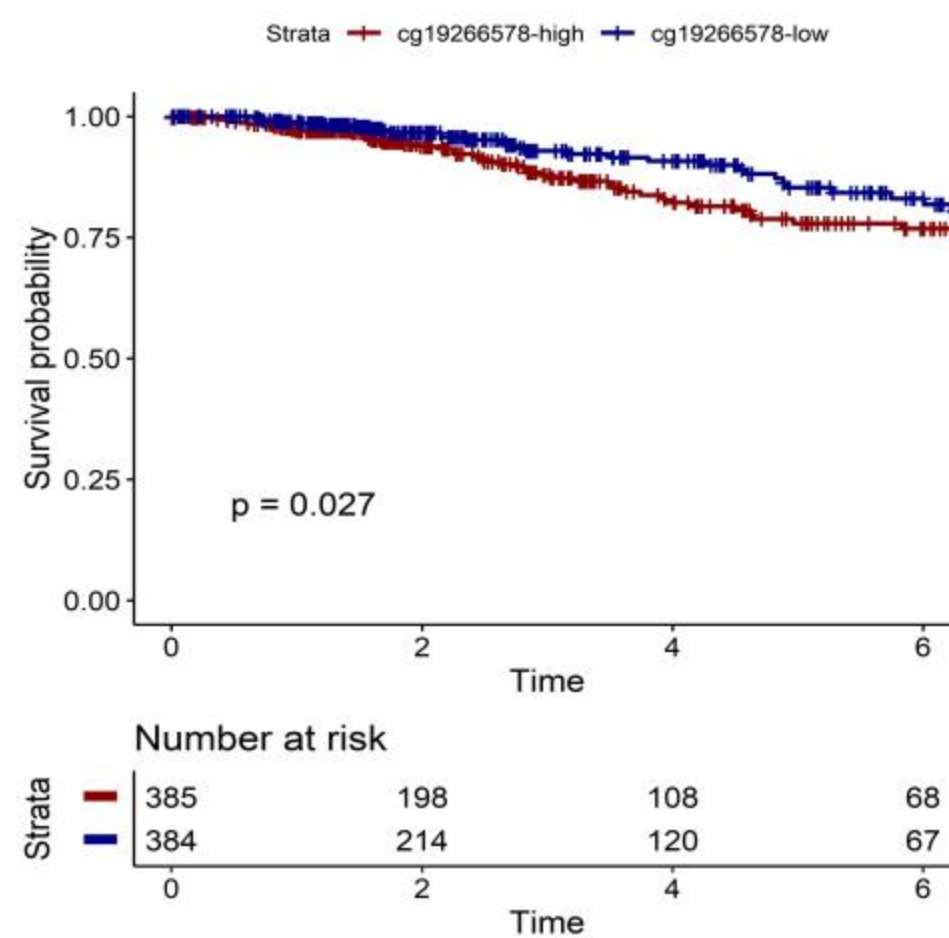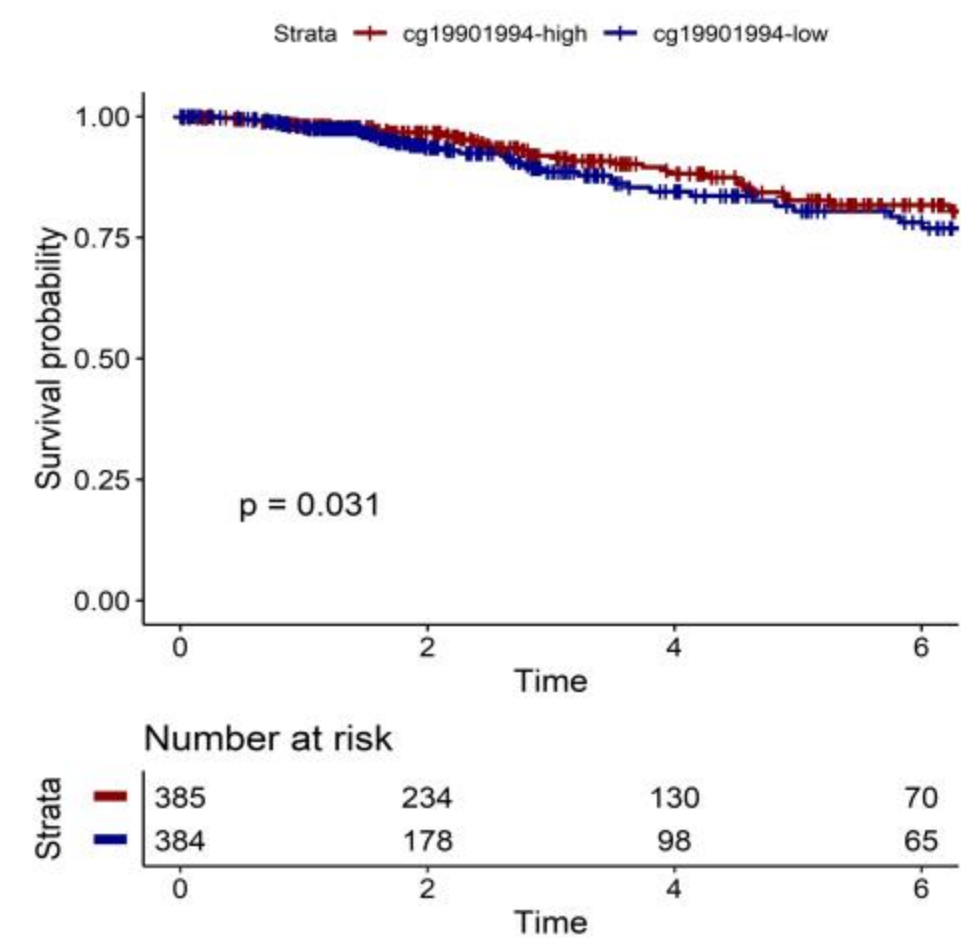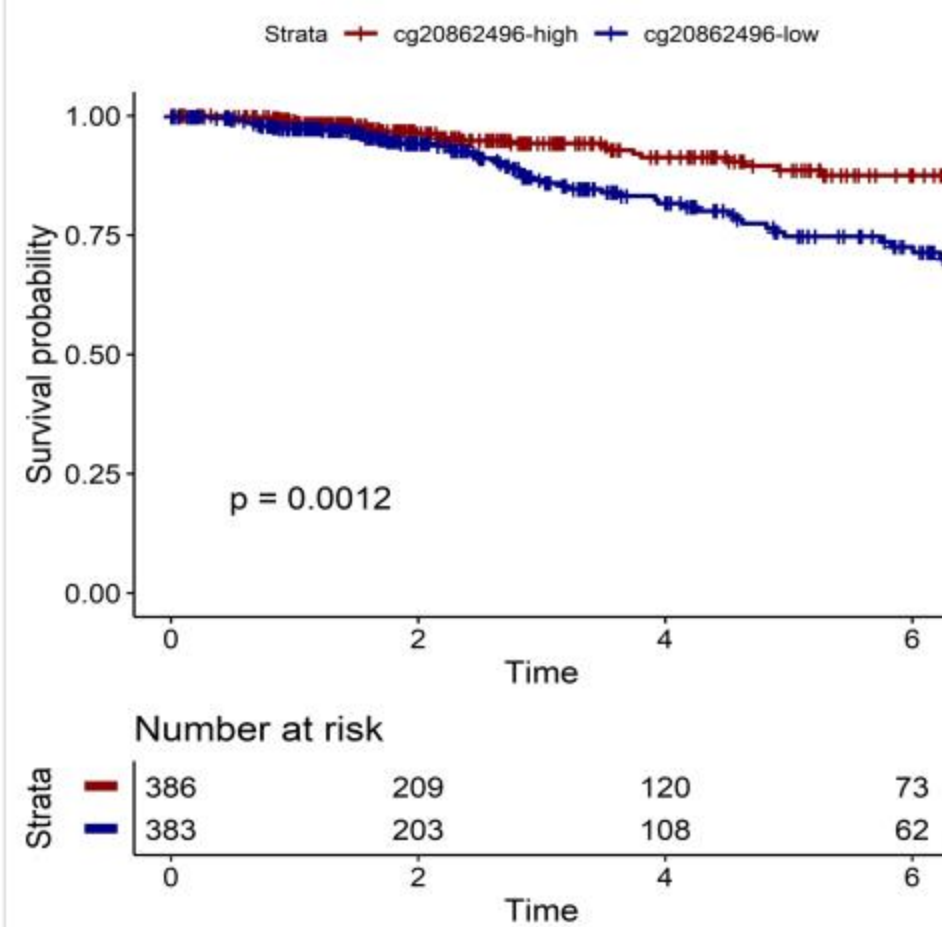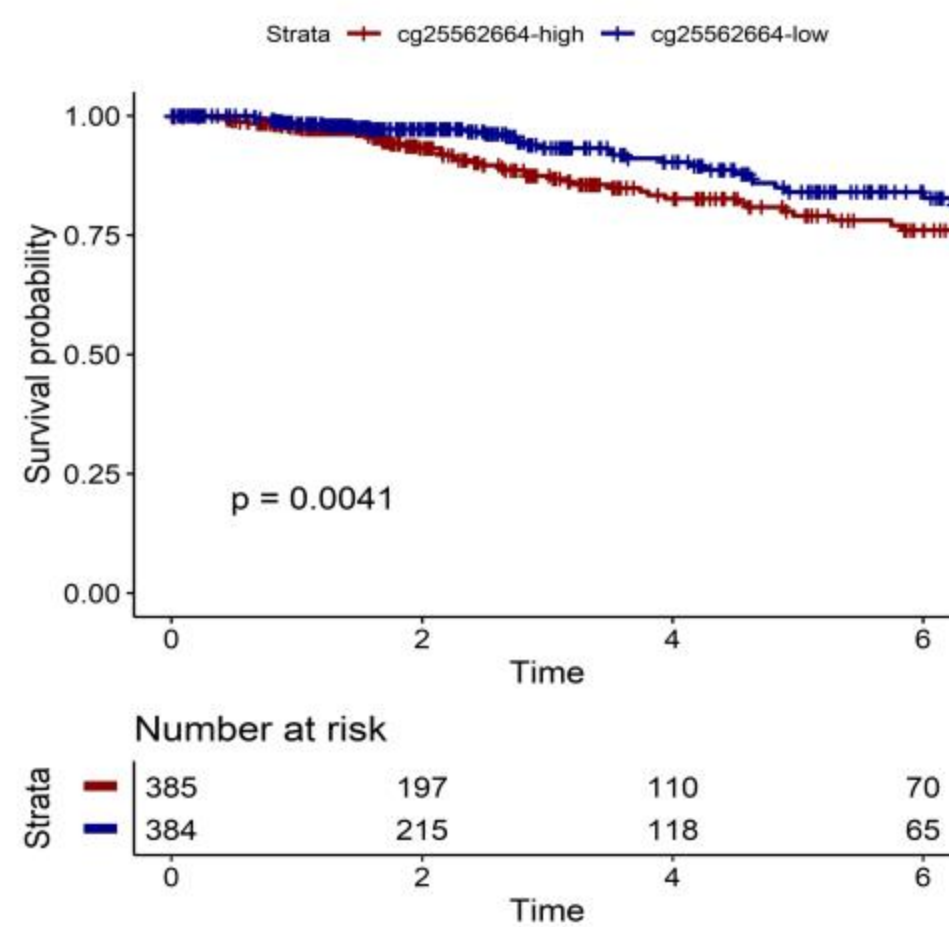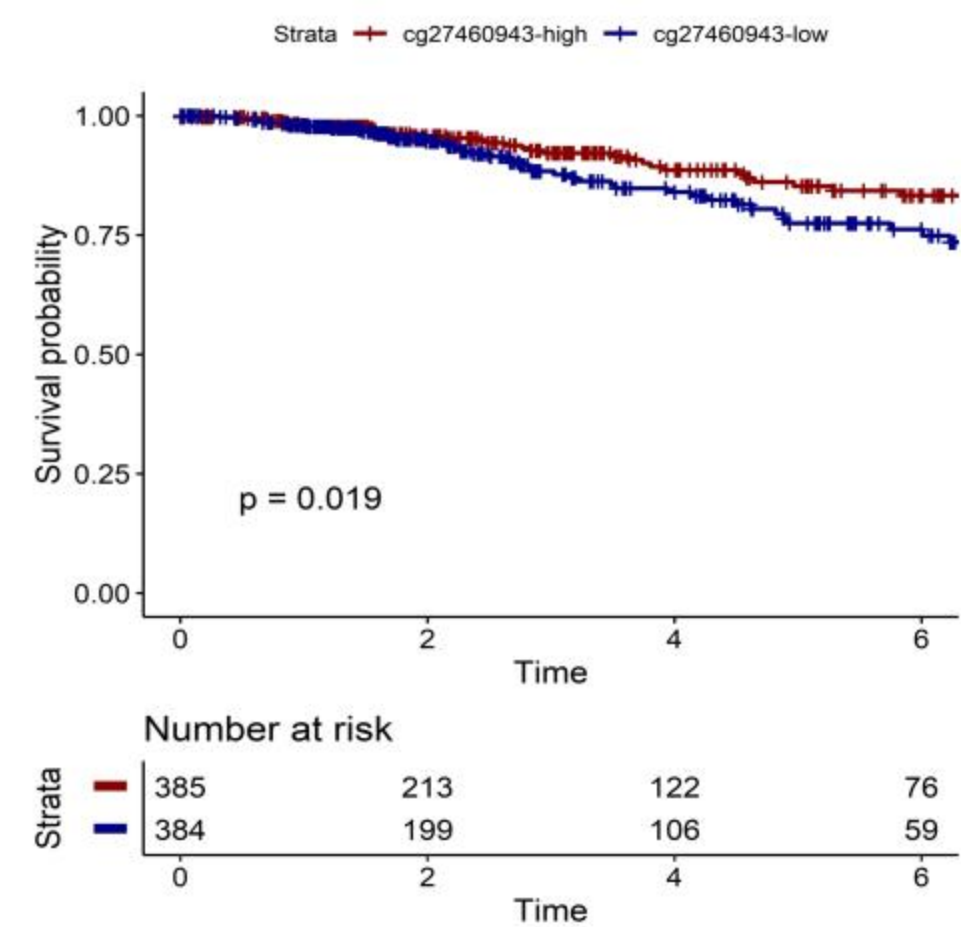

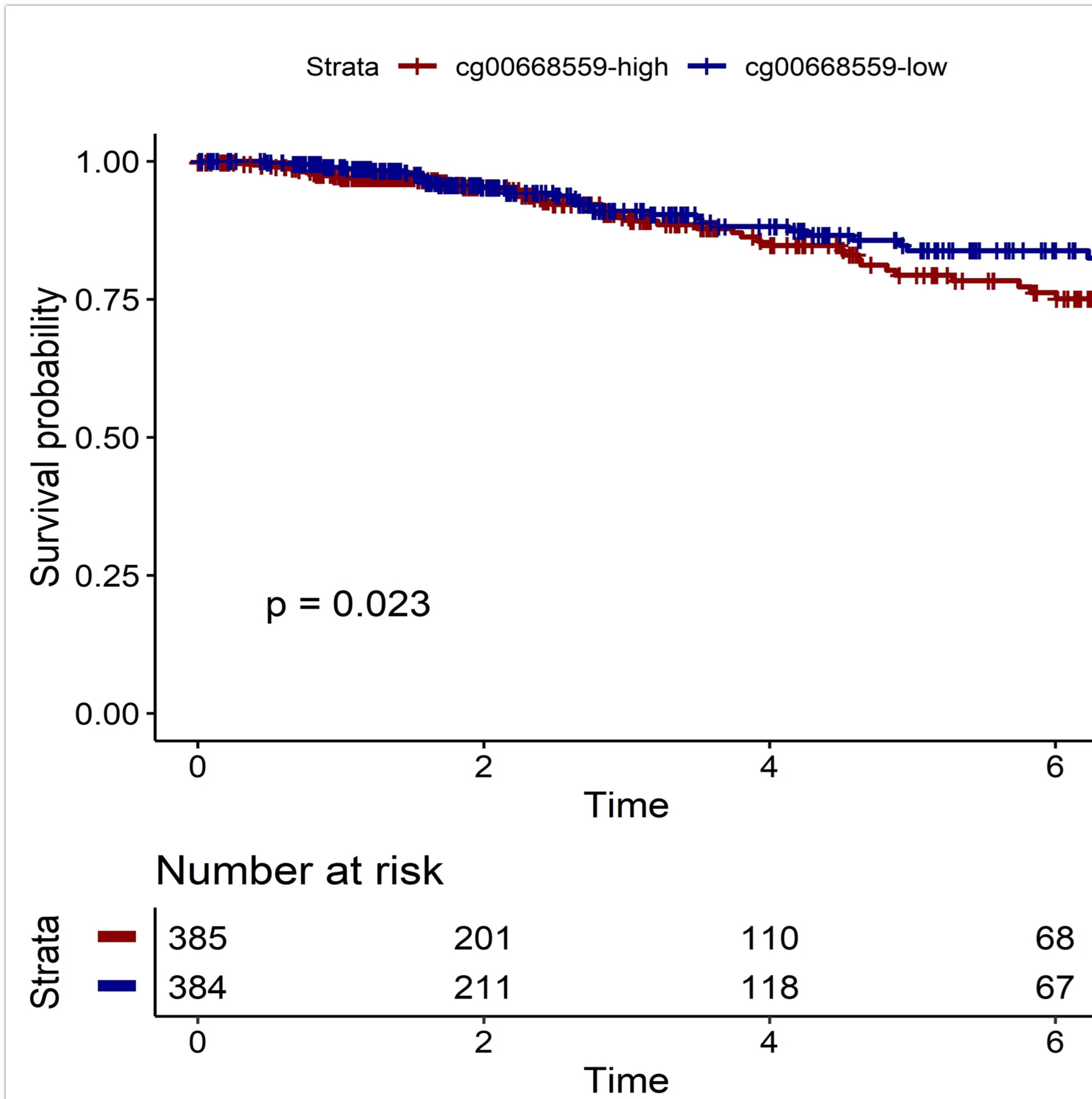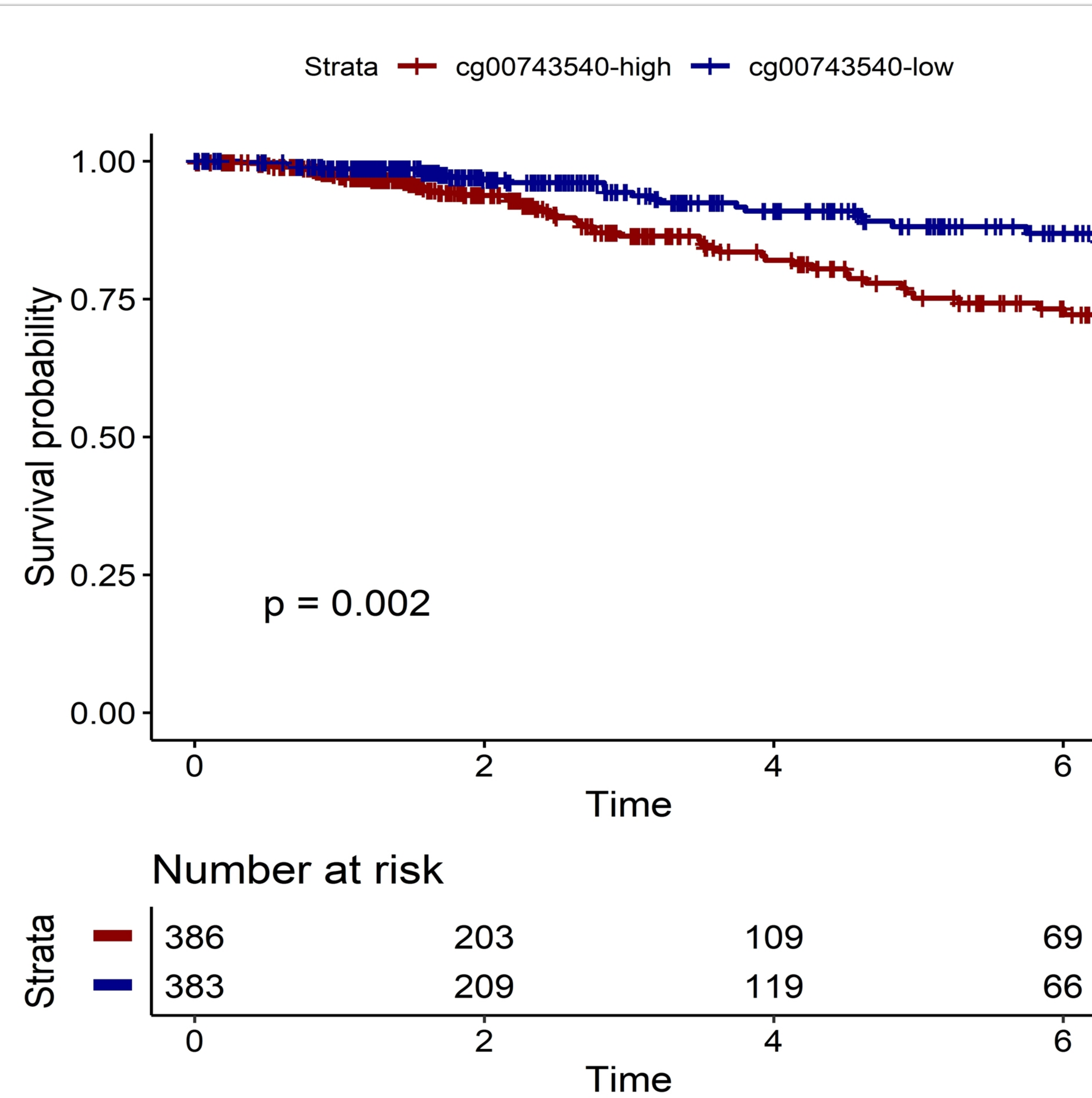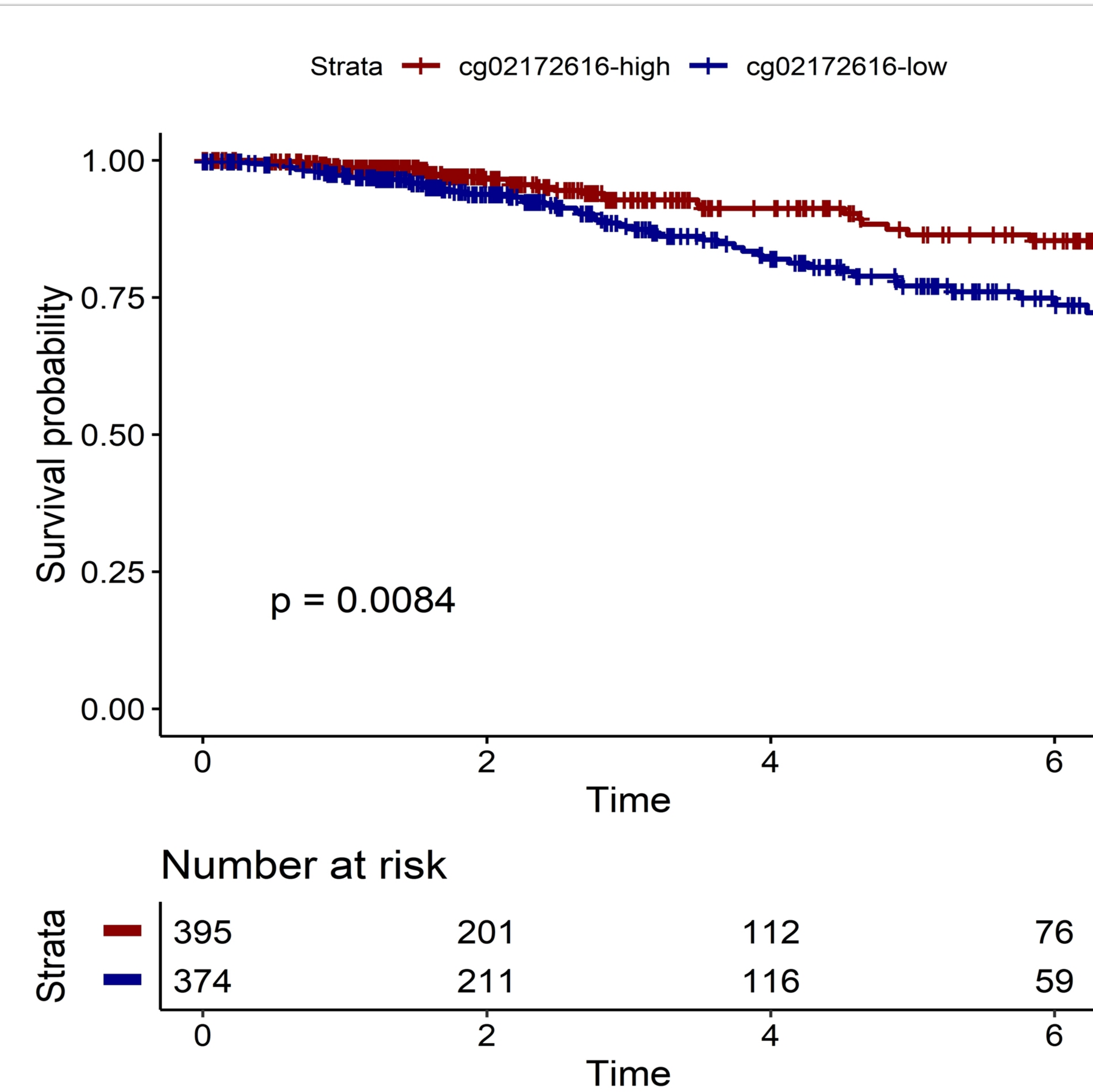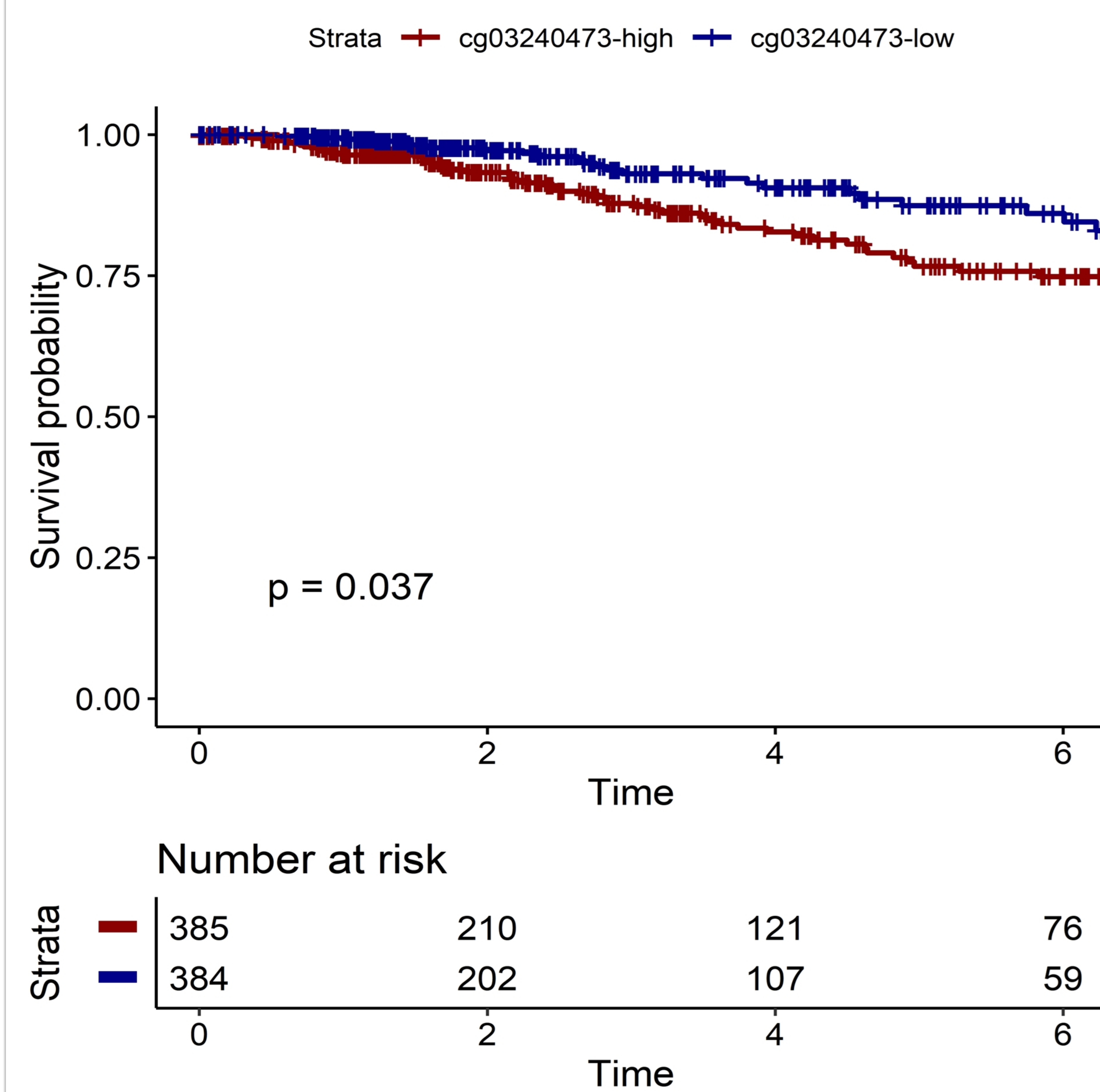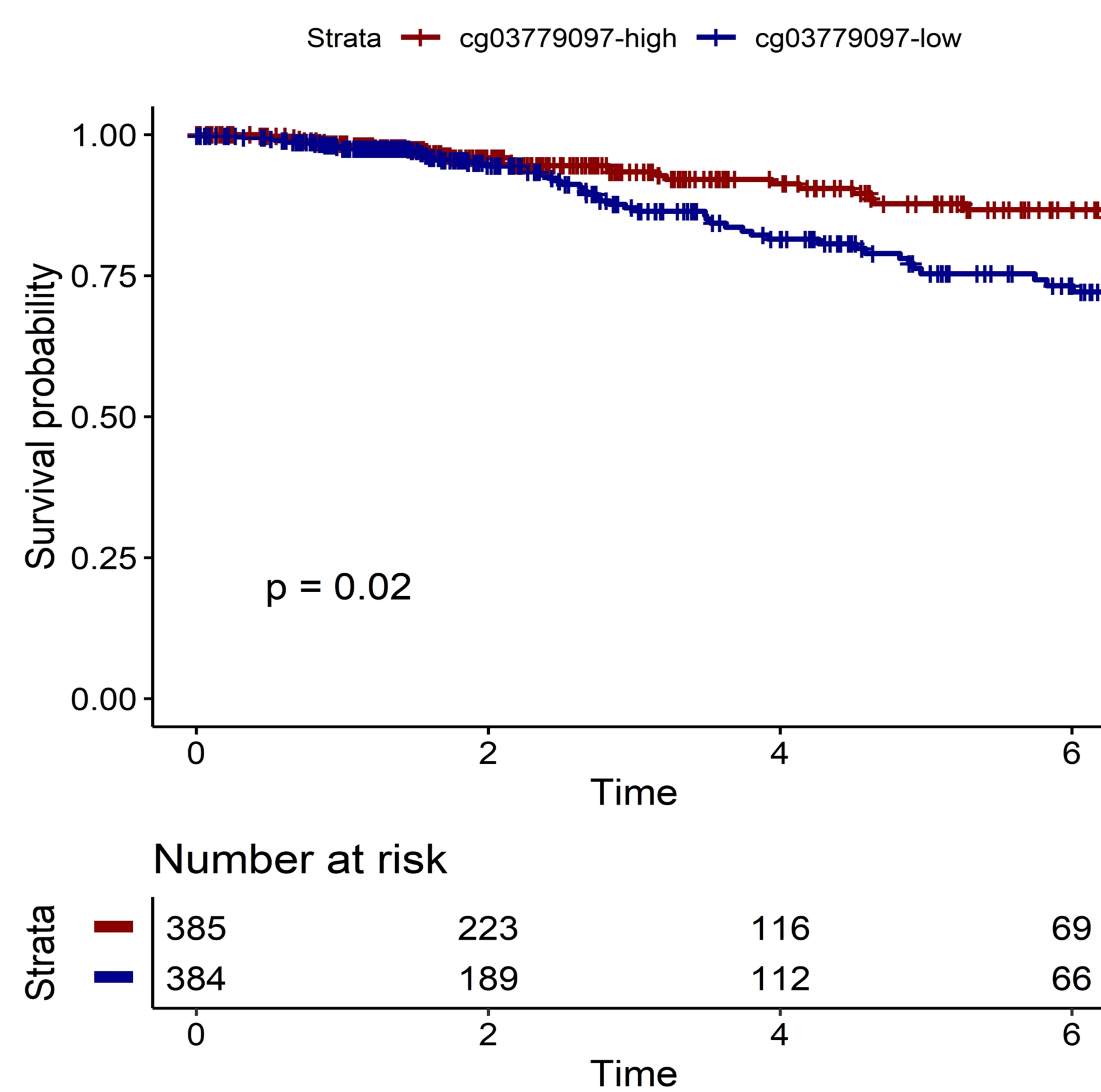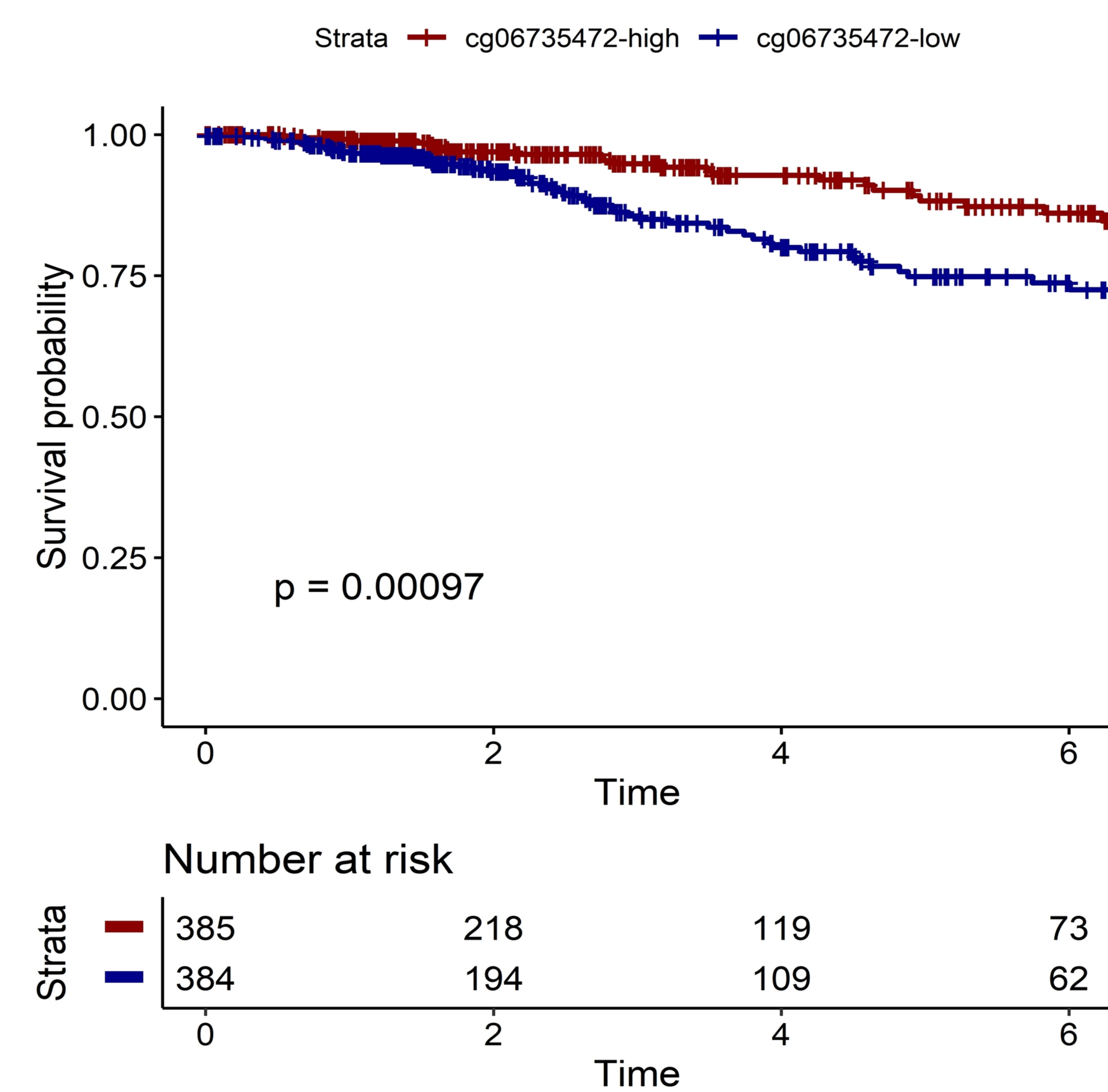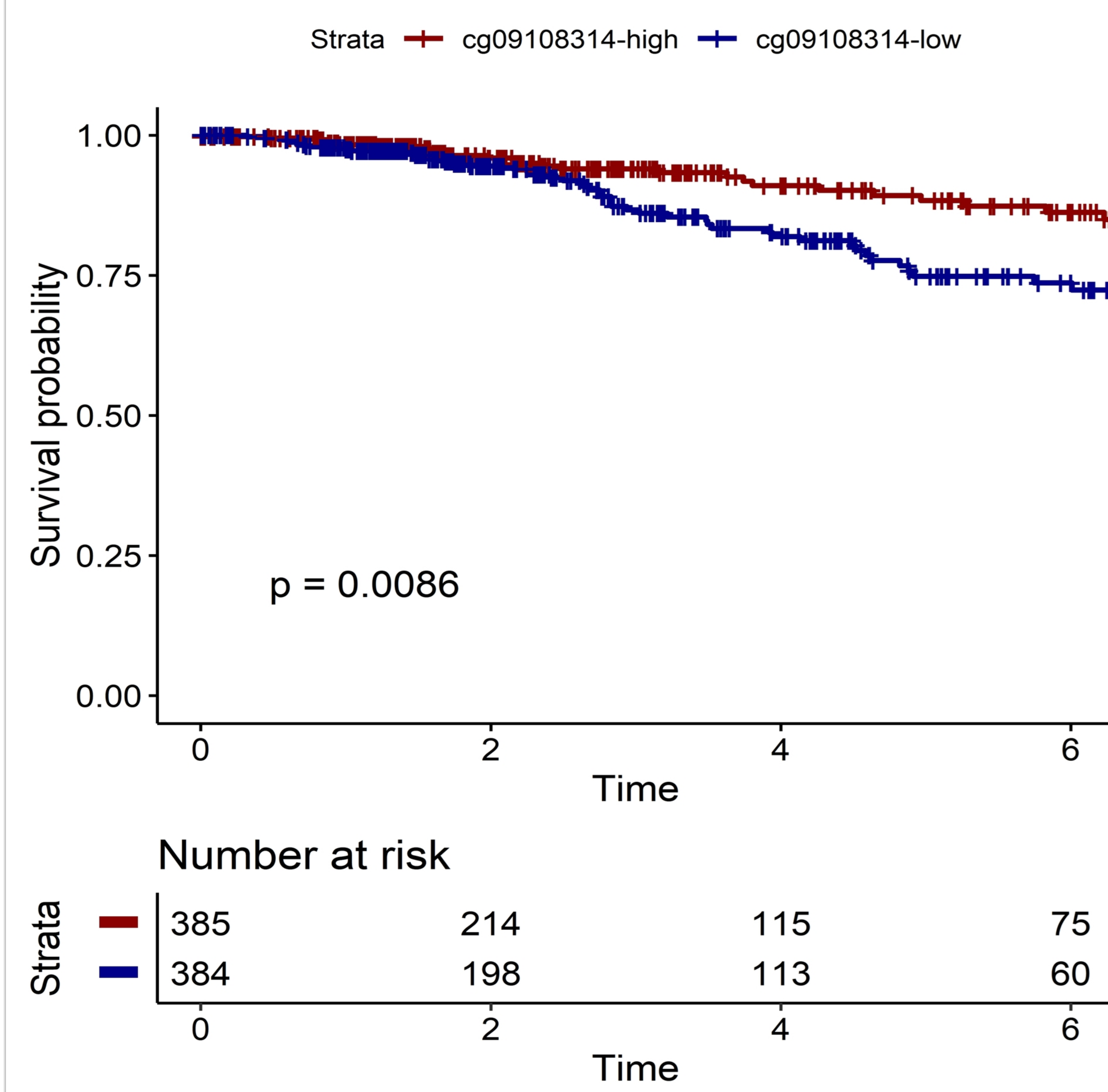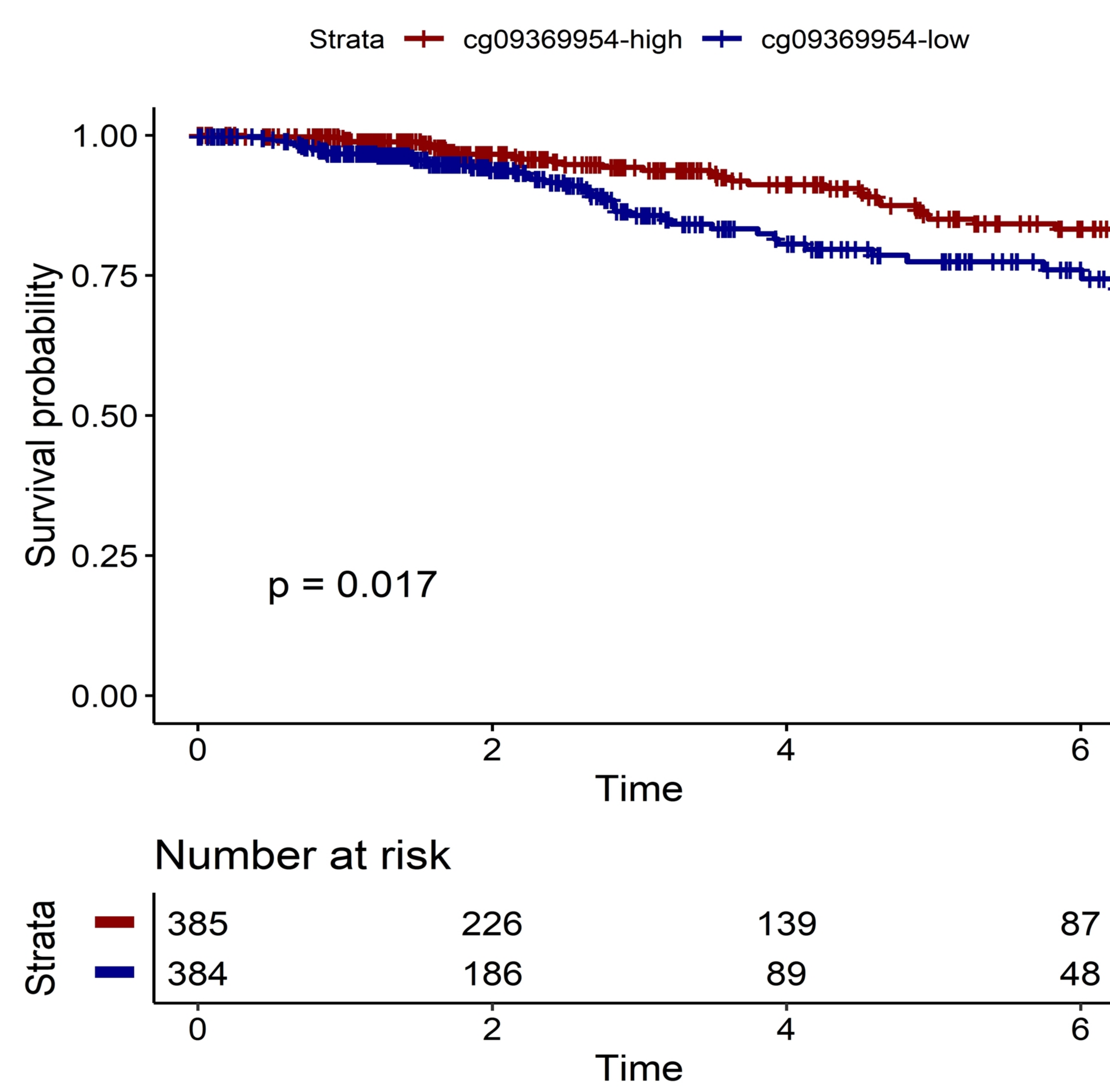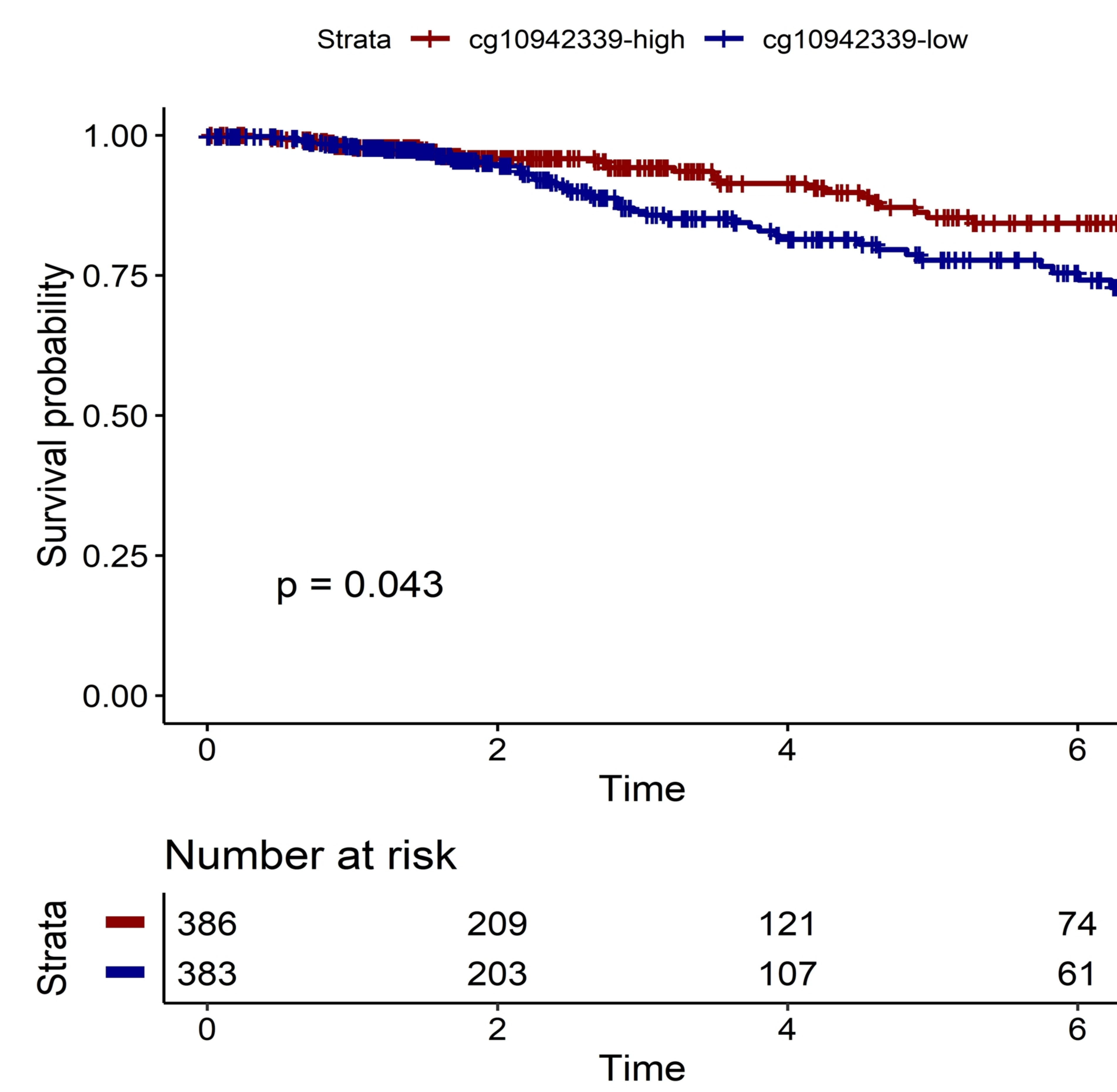

Supplement: Supplementary Figure 1 — Kaplan-Meier plot of overall survival for 18 individual immune methylation sites showed that each immune methylation site could distinguish high-risk and low-risk patients in the TCGA cohort. The log-rank test was used for data analysis. [file DataSheet_1.pdf]
